# Effects of black tea extracts on the quality of cigar tobacco leaves during fermentation

**DOI:** 10.3389/fbioe.2026.1997135

**Published:** 2026-10-30

**Authors:** Yaqi Shi, Tianfei Zheng, Dongfeng Guo, Cunyong Zhang, Jinlong Zhou, Kun Zong, Shaoxuan Ju, Xingjiang Li

**Affiliations:** 1 School of Food and Biological Engineering, Hefei University of Technology, Hefei, China; 2 Technology Center, China Tobacco Anhui Industrial Co. Ltd., Hefei, China

**Keywords:** black tea extracts, cigar tobacco leaves, fermentation, microbial community, sensory quality, volatile aroma compounds

## Abstract

**Background:**

The quality of cigar tobacco leaves (CTLs) is largely determined by fermentation, yet conventional practices suffer from prolonged duration, limited aroma diversity, and residue accumulation. This study evaluated the effects of four black tea extracts (BTEs)—Pu-erh, Keemun, Dianhong, and Dahongpao—on CTL fermentation and elucidated the underlying mechanisms.

**Methods:**

Ultra-high-performance liquid chromatography coupled with quadrupole-orbitrap MS/MS (UHPLC-Orbitrap-MS) was used to characterize the chemical composition of BTEs. High-throughput sequencing was performed to analyze shifts in microbial community structure. Volatile aroma compounds (VACs) were detected via headspace solid-phase microextraction combined with gas chromatography–mass spectrometry (HS-SPME-GC-MS), and sensory evaluation was conducted to assess the fermentation effect.

**Results:**

BTE supplementation significantly restructured the CTL microbial community, increasing Shannon diversity by 20%–40% (TQM: 4.82 ± 0.21 vs. CK: 3.45 ± 0.18, p < 0.05) and enriching potentially beneficial genera such as *Sphingomonas* while suppressing spoilage-associated *Staphylococcus*. Moreover, BTEs markedly increased both the diversity and abundance of VACs. Among the four extracts, Dahongpao and Keemun exhibited the most comprehensive sensory improvements (overall quality: 8.6 ± 0.2 and 8.4 ± 0.2 vs. CK: 5.9 ± 0.2, p < 0.05). Correlation network analysis revealed significant associations among BTE constituents (ferulate, theanine, corilagin), specific ASVs (r > 0.8, q < 0.05), and key VACs.

**Conclusion:**

This study presents a natural strategy for optimizing CTL fermentation, supporting cross-industry integration between the tea and tobacco sectors and providing a technical foundation for producing high-quality cigars.

## Introduction

Cigar quality is largely determined by the fermentation of cigar tobacco leaves (CTLs) (Zhang et al., 2025). Traditional fermentation relies on microbial and enzymatic activities to degrade cellulose, proteins, and alkaloids (e.g., nicotine), thereby reducing harshness and generating characteristic flavors such as cocoa and woody notes (Ning et al., 2023). Nevertheless, this process has significant limitations. First, fermentation is time-consuming, often requiring 1–6 months. Second, flavor diversity remains constrained, as the sensory characteristics depend heavily on tobacco variety and geographical origin, offering limited scope for customization (Zheng et al., 2022a). Third, harmful residues, including nitrosamines and tar, may accumulate or persist when fermentation is incomplete (Wu et al., 2023). Rising consumer demand for cigars with reduced nicotine content and enhanced aromatic profiles is spurring innovation in processing technologies (Yang, 2014). Consequently, the industry continues to explore and develop novel fermentation methods.

Exogenous plant-derived extracts have emerged as effective fermentation modulators by regulating the CTL microecosystem. For instance, Hu et al. (2024) demonstrated that fermenting CTLs with citrus flavonoids reduced irritation and improved aroma richness. Yao et al. (2025) found that tobacco flower bud extract reshaped the microbial community, suppressing harmful microbes while promoting aroma compound synthesis. Li et al. (2025) developed an exogenous additive from *Psoralea corylifolia* fruit that enhanced the bean-like aroma and honey sweetness of CTLs.

Tea, a globally consumed beverage, is rich in bioactive compounds including polyphenols, polysaccharides, theanine, and caffeine (Luo et al., 2024a; Zhang et al., 2023). These components exhibit antioxidant, antibacterial, and microbial metabolism-regulating properties and have been widely applied in food fermentation and agricultural preservation. Tea polyphenols scavenge free radicals generated during fermentation, mitigating oxidative damage (Luo et al., 2024b). Tea polysaccharides modulate the microbial environment and promote beneficial bacterial proliferation (Zeng et al., 2025). Theanine can synergistically interact with tobacco flavor compounds to improve aroma harmony (Lin et al., 2023). Given these multiple bioactivities, tea extracts hold considerable potential for enhancing CTL fermentation quality. While individual tea-derived compounds (e.g., EGCG, theanine) have demonstrated bioactivities, crude tea extracts offer practical advantages including synergistic interactions among multiple components, lower production costs, and greater alignment with consumer preferences for ‘natural’ additives. Moreover, the complex chemical matrix of whole tea extracts may exert more comprehensive modulatory effects on the fermentation ecosystem than single compounds.

This study aimed to evaluate the effects of four BTEs—Pu-erh, Keemun, Dianhong, and Dahongpao—on CTL fermentation and to clarify the underlying mechanisms. We characterized BTE chemical components using UHPLC-Orbitrap-MS, analyzed microbial communities via high-throughput sequencing, detected volatile aroma compounds (VACs) by HS-SPME-GC-MS, and assessed fermentation effect through sensory evaluation. Correlation analyses were performed to reveal links among BTE bioactive components, microbial taxa, and VACs. This work provides technical support for innovating CTL fermentation and promotes cross-industry synergy between the tea and tobacco sectors.

## Materials and methods

### Materials and reagents

Four black tea varieties—Pu-erh (PE), Keemun (QM), Dianhong (DH), and Dahongpao (HP)—were purchased from a local supermarket in Hefei, China. Their origins and processing methods are listed in Table 1. CTLs (Yunxue No. 1 filler tobacco) from Yunnan were used for fermentation. HPLC-grade acetonitrile (ACN), isopropanol (IPA), and acetic acid were obtained from Thermo Fisher Scientific (Waltham, MA, United States). All other reagents were of analytical grade.

**TABLE 1:** Origin and treatment of black tea samples.

Samples	Black tea	Treatment method	Place of origin
QM	Keemun black tea	Water extraction	Anhui
HP	Da hong pao	Water extraction	Fujian
DH	Dianhong	Water extraction	Yunnan
PE	Pu-erh	Water extraction	Yunnan

### Preparation of black tea extracts

BTEs were prepared by hot water extraction. Briefly, 10 g of each tea sample was extracted with 200 mL of boiling distilled water for 30 min under constant stirring. The extracts were cooled to room temperature and centrifuged at 7,000 × g and 4 °C for 10 min to remove insoluble residues. The resulting supernatant was filtered through a 0.22 μm filter to eliminate native microbial communities potentially associated with the tea samples. The obtained BTEs were stored at −20 °C until use.

## Analysis of chemical components in black tea extracts

Chemical profiling of BTEs was performed using a UHPLC system (Vanquish, Thermo Fisher Scientific) coupled with an Orbitrap Exploris 120 mass spectrometer (Thermo Fisher Scientific). Separation was conducted on a Phenomenex Kinetex C18 column (2.1 mm × 50 mm, 2.6 μm) with mobile phase A (water with 0.01% acetic acid) and mobile phase B (IPA:ACN = 1:1, v/v) under gradient elution (0–2 min, 5% B; 2–10 min, 5%–95% B; 10–12 min, 95% B; 12–14 min, 95%–5% B; 14–16 min, 5% B) at 40 °C, with a flow rate of 0.4 mL/min and injection volume of 5 μL. Mass spectra were acquired in positive (3.5 kV) and negative (−2.5 kV) electrospray ionization modes over a scan range of 100–1700 m/z, with MS/MS fragmentation at 10–30 eV NCE. Raw data were processed using ProteoWizard and an in-house R script based on XCMS. Metabolite identification was performed at three confidence levels: Level 1 (authentic standards, retention time and MS/MS match), Level 2 (MS/MS spectral match to library with >80% similarity score), and Level 3 (accurate mass match only). Compounds discussed in the result section (e.g., ferulate, theanine, corilagin, EGCG) were identified at Level 1.

## Fermentation of cigar tobacco leaves with black tea extracts

Fermentation of CTLs was carried out as described in previous studies (Zheng et al., 2022c). To determine the optimal BTE application concentration, a preliminary dose–response experiment was conducted using the QM extract (Keemun black tea) at four concentrations: 10%, 20%, 30%, and 40% (v/w, diluted 10-fold BTE solution sprayed onto CTLs). The 30% concentration yielded the highest overall sensory score (8.1 ± 0.3), outperforming 10% (6.2 ± 0.4), 20% (7.3 ± 0.3), and 40% (7.5 ± 0.4, with excessive moisture causing localized mold growth in preliminary trials). Based on these results, 30% concentration was selected because it achieved the highest sensory score without any visible mold growth. Accordingly, each BTE was diluted 10-fold with sterile water and sprayed onto CTLs at 30% (v/w), then fermented in a chamber at 40 °C and 85% relative humidity for 7 days. During the main fermentation experiment, all samples were visually inspected daily, and no mold contamination was observed in any group. Traditional spontaneous fermentation is time-consuming, often requiring 1–6 months. This study investigates a 7-day BTE-assisted fermentation as an accelerated alternative, and evaluates whether the resulting product quality is comparable to that of conventionally fermented CTLs. For each treatment group, three independent fermentation batches were performed on separate days using separate fermentation vessels (each containing 500 g of CTLs). Each batch was treated as one biological replicate. Samples were labeled TQM (QM), THP (HP), TPE (PE), and TDH (DH), CTLs treated with water only as the control (CK). After fermentation, samples were freeze-dried and stored at −20 °C for subsequent analysis.

## Analysis of volatile aroma compounds

VACs were analyzed using HS-SPME-GC-MS, three samples were taken from each batch and averaged to obtain one value per batch (n = 3 biological replicates). Freeze-dried CTLs were ground to a powder; 1 g of powder was placed in a 20 mL headspace vial with 10 μL of 2-octanol (10 mg/L) as internal standard. Volatiles were extracted using a 50/30 μm DVB/CAR/PDMS fiber at 60 °C for 30 min and analyzed on a GC-TOFMS system (Pegasus BT, LECO) equipped with a DB-Wax column (30 m × 0.25 mm × 0.25 μm). Helium was the carrier gas (1 mL/min). The injector temperature was 250 °C, and the oven program was: 40 °C for 2 min, ramp at 10 °C/min to 250 °C, hold 5 min. Mass spectra were acquired in EI mode at −70 eV over a 20–400 m/z range. Data were processed using Chroma TOF 4.3X software and the NIST library (Kind et al., 2009). The relative content of each volatile compound was calculated as the peak area ratio to the internal standard (2-octanol) and expressed as 2-octanol equivalents (μg/g dry weight). This semi-quantitative approach assumes a response factor of 1.0 for all compounds, which may introduce bias for compounds with significantly different ionization efficiencies.

### Analysis of microbial communities

Microbial community analysis was performed via amplicon sequencing; one sample was taken from each of the three independent batches (n = 3 biological replicates). Microorganisms were collected by washing CTLs with sterile saline. Specifically, 5 g of CTLs were homogenized in 100 mL of sterile saline and agitated at 220 rpm for 4 h. The 4 h shaking duration was selected based on preliminary tests showing that shorter durations (30 min to 2 h) yielded insufficient microbial biomass for reliable DNA extraction (DNA concentration < 5 ng/μL), likely due to the waxy and hydrophobic surface of CTLs. The suspension was filtered through sterile gauze and centrifuged at 7,000 × g for 15 min. The microbial pellets were stored at −20 °C until DNA extraction. Total genomic DNA was extracted using the DNeasy PowerSoil Kit (QIAGEN) according to the manufacturer’s instructions. DNA quality and concentration were assessed via NanoDrop ND-1000 and agarose gel electrophoresis. The V3–V4 region of the bacterial 16S rRNA gene was amplified with primers 341F (5′-ACT​CCT​ACG​GGA​GGC​AGC​AG-3′) and 806R (5′-GGACTACHVGGGTWTCTAAT-3′). PCR products were purified, quantified, and sequenced on an Illumina NovaSeq PE250 platform (2 × 250 bp). Raw reads were filtered to remove those with a quality score < 20 and length < 200 bp. After denoising and chimera removal, an average of 45,632 ± 8,214 high-quality reads per sample were obtained, with an average sequence length of 253 bp (Callahan et al., 2016). Taxonomic classification was performed and analyzed using QIIME2 (Bolyen et al., 2019).

### Sensory evaluation

Sensory evaluation was conducted by a panel of five professional tasters (4 male, 1 female; age range: 35–52 years; all with 10–20 years of experience in cigar sensory assessment and regular cigar smoking habits). All panelists were trained according to standard tobacco industry protocols, each of the five panelists evaluated each batch three times, and the mean of all panelist-batch combinations was used for statistical analysis (n = 5 panelists × 3 batches). Fermented CTLs were hand-rolled into cigars (50 mm length, 10 mm diameter) and conditioned at 20 °C and 68% ± 1% relative humidity for 48 h. Each sample was coded with a random three-digit number and presented to panelists in randomized order to minimize order effects. Panelists blindly scored five attributes on a 1–10 scale: aroma richness, irritancy (lower score = less irritation), smoke smoothness, aftertaste, and overall quality. The scoring criteria for each attribute were defined as follows: aroma richness (1–3: weak and simple; 4–6: moderate with some complexity; 7–10: rich and highly complex); irritancy (1–3: mild; 4–6: moderate; 7–10: harsh and pungent); smoke smoothness (1–3: rough; 4–6: moderately smooth; 7–10: very smooth and mellow); aftertaste (1–3: short and unpleasant; 4–6: moderate duration; 7–10: long-lasting and pleasant); overall quality (1–3: poor; 4–6: acceptable; 7–10: excellent). Each sample was evaluated three times by each panelist, and mean scores were calculated. Inter-rater agreement was assessed using Kendall’s W coefficient of concordance, which yielded a value of 0.84 (p < 0.01), indicating good consensus among panelists.

### Statistical analysis

All statistical analyses were performed using R. For heatmap generation (Figures 1, 2), data were log2-transformed and row-scaled (z-score normalization) to visualize relative differences among samples. Spearman’s correlation coefficients were calculated among the top 36 BTE constituents, top 30 ASVs, and top 30 VACs. Correlation networks were constructed using Gephi 0.9.2 with thresholds of |r| > 0.8 and p < 0.05. Sensory data were analyzed using linear mixed-effects models (lme4 package in R) with treatment as a fixed effect and panelist as a random effect to account for repeated ratings from the same individual. Pairwise comparisons were performed using Tukey’s HSD test. Batch-level means were used as the unit of analysis for biological replication. Significance was set at α = 0.05. Normality and homogeneity of variances were confirmed using Shapiro–Wilk and Levene’s tests, respectively. To mitigate false discovery due to multiple hypothesis testing, the Benjamini–Hochberg false discovery rate (FDR) correction was applied to all p-values derived from correlation analyses, with a significance threshold of q < 0.05. For correlation analysis between BTE constituents and microbial ASVs, the four BTE treatment groups (TQM, THP, TPE, TDH) were used as independent observations (n = 4), with each BTE’s chemical profile paired to the corresponding treatment’s microbial community data. The water control (CK) was excluded from this analysis because it did not receive any BTE. For correlation analysis between microbial ASVs and VACs, all five groups (including CK) were used (n = 5), with ASV relative abundances paired to VAC relative abundances within each group. Spearman’s correlation coefficients were calculated, and p-values were adjusted using the Benjamini–Hochberg FDR method (q < 0.05). A stringent |r| > 0.8 threshold was applied. It should be emphasized that these correlations represent statistical associations, not causal relationships, and the small sample size (n = 4 or 5) limits statistical power.

**FIGURE 1:**
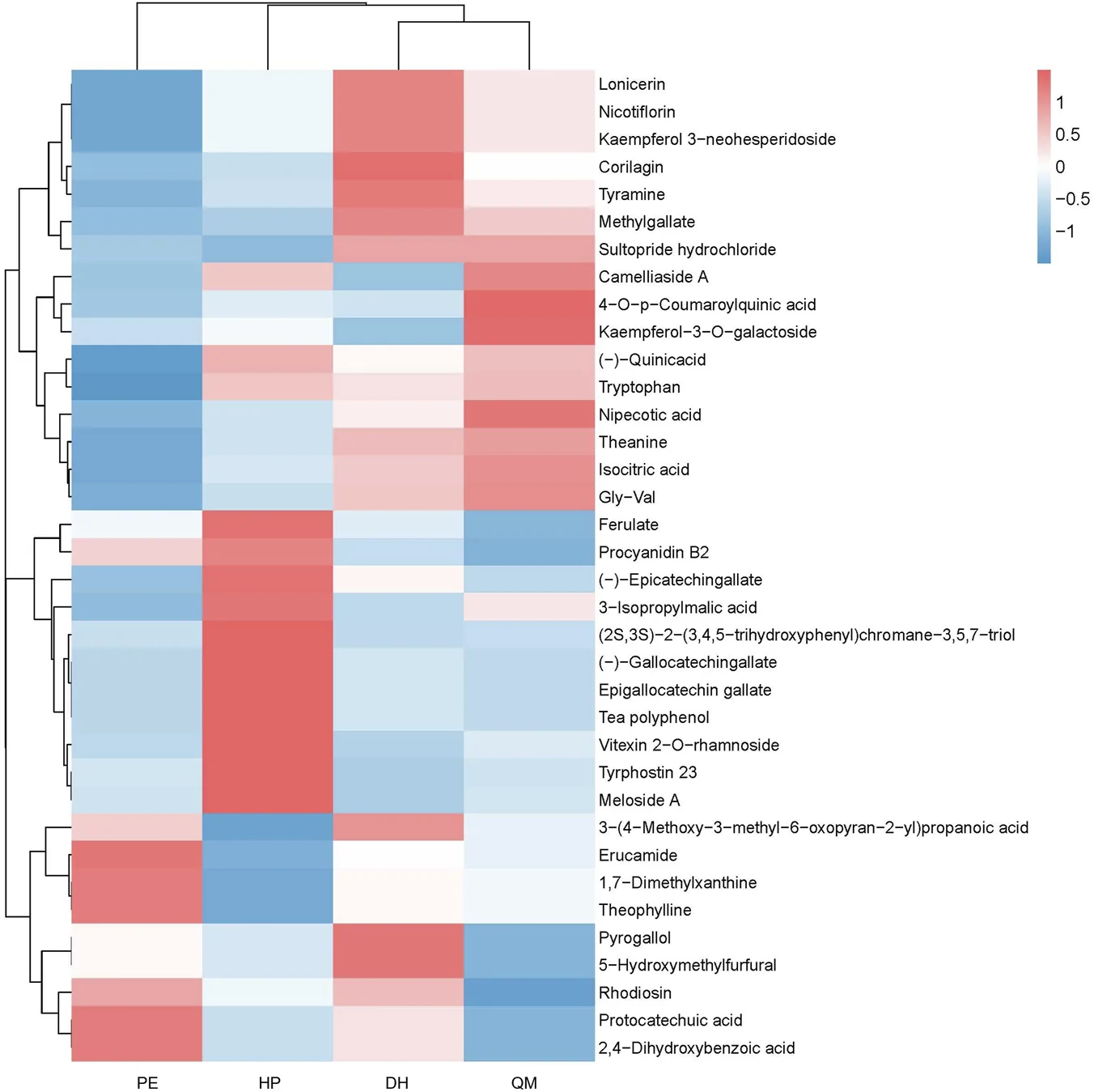
Main constituents (top 36 by relative abundance) in black tea extracts, the mean of three biological replicates per tea extract is shown.

**FIGURE 2:**
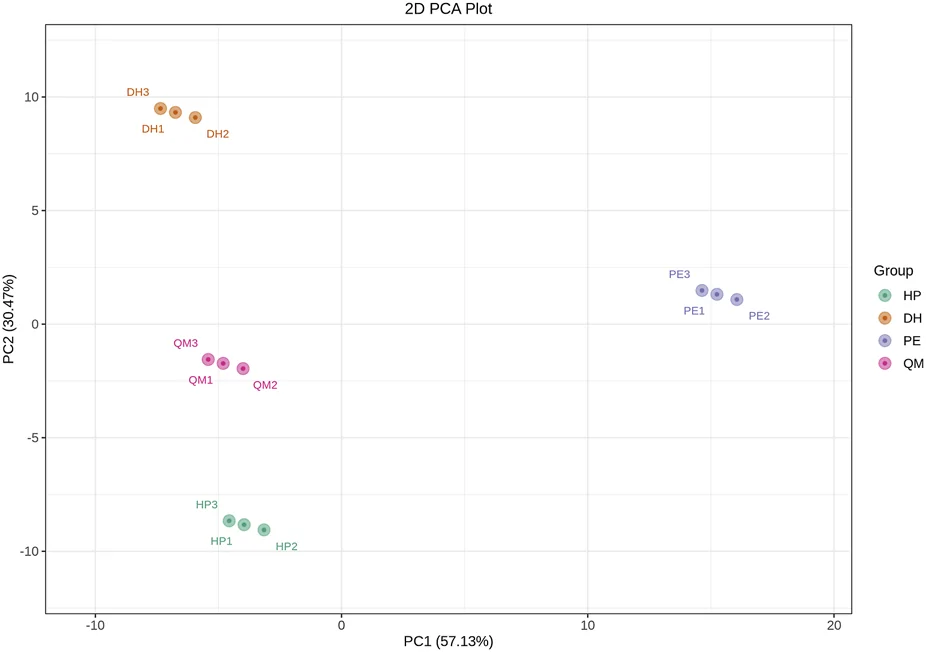
Principal component analysis (PCA) of the four black tea extracts based on UHPLC-Orbitrap-MS metabolite profiles (n = 3 biological replicates per extract). Data were mean-centered and Pareto-scaled prior to analysis. Ellipses represent 95% confidence intervals around group centroids. PC1 and PC2 explain 57.13% and 30.47% of the total variance, respectively.

## Results

### Chemical composition of black tea extracts

UHPLC-Orbitrap-MS identified hundreds of compounds across the four BTEs; the top 36 by relative abundance are shown in Figure 1. Major components included tea polyphenols, theanine, caffeine, flavonoids, organic acids, and alkaloids, with substantial variation among extracts. The QM extract was rich in compounds consistent with the umami character of Keemun black tea (Jiang et al., 2025). The HP extract (Dahongpao) contained lonicerin, nicotiflorin, kaempferol 3-neohesperidoside, corilagin, tyramine, methylgallate, pyrogallol, and 5-hydroxymethylfurfural—the latter a Maillard reaction product contributing caramel-like notes (Wang J. et al., 2022). The DH extract (Dianhong) was abundant in ferulate, procyanidin B2 (−)-epicatechin gallate, 3-isopropylmalic acid (-)-gallocatechin gallate, epigallocatechin gallate (EGCG), vitexin 2-O-rhamnoside, tyrphostin 23, and meloside A. EGCG was particularly abundant, underscoring the strong antioxidant capacity of Dianhong extract (Peng et al., 2025). The PE extract (Pu-erh) featured erucamide, 1,7-dimethylxanthine (paraxanthine), theophylline, rhodiosin, protocatechuic acid, and 2,4-dihydroxybenzoic acid. Theophylline, a methylxanthine alkaloid, is known for its bronchodilatory and metabolic effects (Jia et al., 2024). PPrincipal component analysis (Figure 3) showed clear separation among the four extracts, with PC1 and PC2 explaining 57.13% and 30.47% of the total variance, respectively (cumulative: 87.60%), confirming distinct chemical profiles that may underlie their differential effects on fermentation.

**FIGURE 3:**
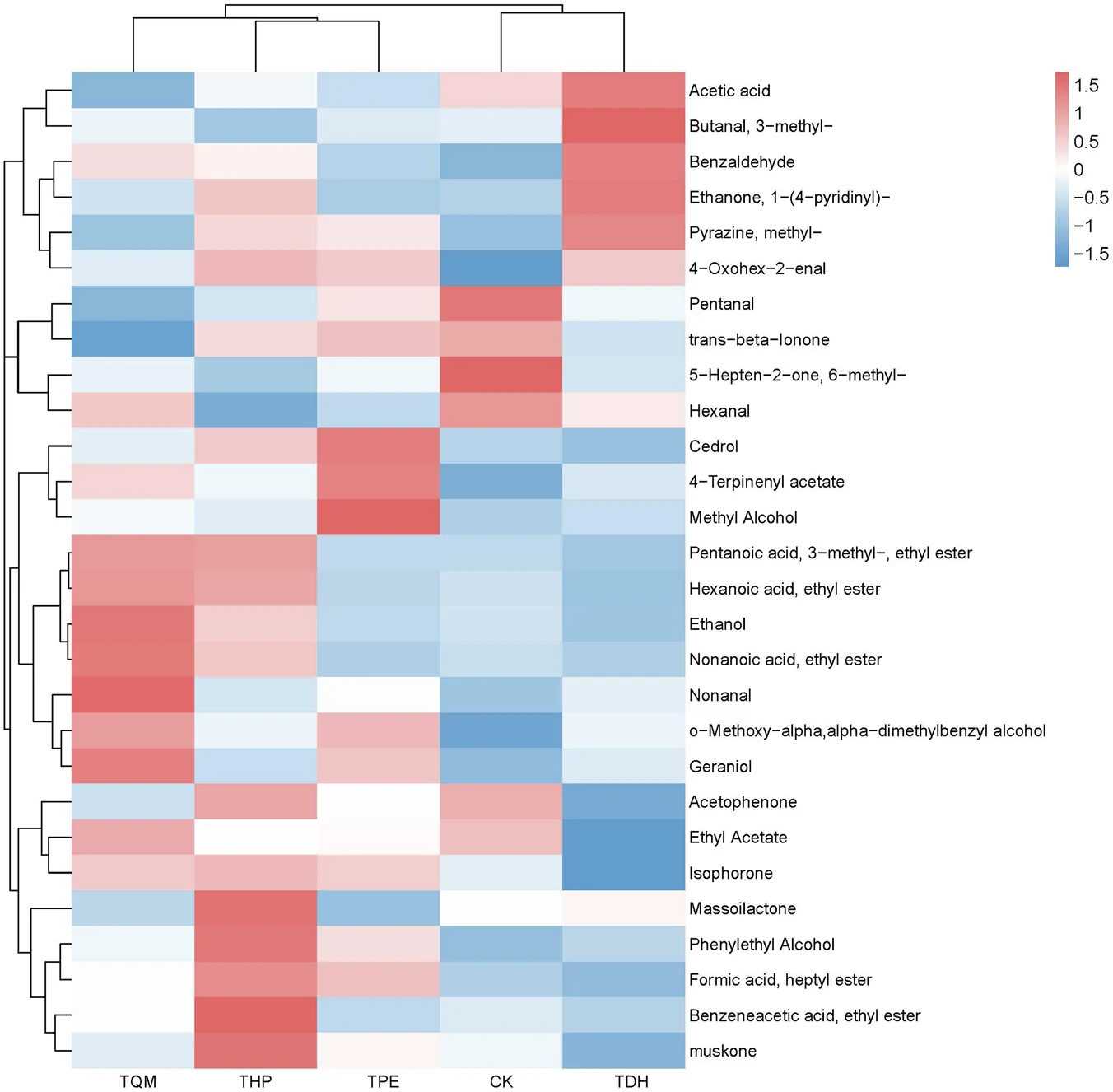
Main volatile aroma compounds in cigar tobacco leaves, the mean of three independent fermentation batches per treatment is shown. Hierarchical clustering was performed using Euclidean distance and Ward’s linkage method.

### Volatile aroma compound profiles

HS-SPME-GC-MS detected hundreds of volatiles in fermented CTLs; the top 28 compounds are presented in Figure 2. These included aldehydes, ketones, alcohols, esters, pyrazines, and terpenoids-key contributors to cigar aroma (Zheng et al., 2022a). In the CK group, the most abundant VACs were trans-β-ionone, pentanal, triisobutyl phosphate, pyridine, 6-methyl-5-hepten-2-one, and hexanal, primarily derived from carotenoid and fatty acid degradation, which may contribute to harshness and limited flavor complexity. In TQM, dominant VACs included ethyl acetate, isophorone, hexanoic anhydride, 3-methylpentanoic acid ethyl ester, hexanoic acid ethyl ester, benzoic acid ethyl ester, ethanol, nonanoic acid ethyl ester, nonanal, and geraniol. Esters such as ethyl acetate and hexanoic acid ethyl ester impart fruity and floral notes, while geraniol contributes a rose-like aroma. In THP, key VACs included phenylethyl alcohol, formic acid heptyl ester, benzeneacetic acid ethyl ester, and muskone—the latter contributing a creamy, sweet note (Scognamiglio et al., 2012). In TDH, abundant VACs included methyl-pyrazine (nutty, roasted), benzaldehyde (almond-like), 1-(4-pyridinyl)-ethanone, 3-methyl-butanal, and acetic acid (Cemin et al., 2022; Takakura et al., 2022). In TPE, major VACs were cedrol (woody, camphoraceous), 4-terpinenyl acetate, and methyl alcohol (Wang S. et al., 2022; Yang et al., 2024). The number and relative abundances of VACs were significantly higher in all BTE-treated groups than in CK (p < 0.05), with TQM showing the greatest diversity, followed by THP. These results indicate that BTEs altered the volatile aroma profiles of fermented CTLs.

### Effects of black tea extracts on microbial communities

To assess the overall impact of BTEs on microbial community diversity, α-diversity indices were calculated for each group. The Shannon diversity index was significantly higher in all BTE-treated groups compared to CK (p < 0.05, Kruskal–Wallis test), with the TQM group showing the highest diversity (Shannon = 4.82 ± 0.21), followed by THP (4.61 ± 0.19), TDH (4.38 ± 0.22), and TPE (4.15 ± 0.20), compared to CK (3.45 ± 0.18) (Table 2). Similarly, the Chao1 richness estimator revealed significantly greater species richness in BTE-treated groups than in CK (p < 0.05), indicating that tea extracts promoted a more diverse microbial ecosystem. Beta diversity analysis based on Bray–Curtis distances and visualized by principal coordinate analysis (PCoA) revealed distinct clustering of the four BTE-treated groups and CK along the first two principal coordinates (PCo1: 38%, PCo2: 27.2% of variance) (Figure 4). PERMANOVA based on Bray–Curtis distances confirmed significant differences among the five groups (R^2^ = 0.38, p < 0.001). Pairwise comparisons with FDR adjustment showed that all four BTE-treated groups differed significantly from CK (p < 0.05), and that TQM, THP, TDH, and TPE were also significantly different from each other (p < 0.05), with the exception of the TQM–THP pair (p = 0.07), which showed marginal separation. These results indicate that each BTE induced a distinctive restructuring of the CTL microbial community. The sensory panel size (n = 5) is relatively small, which may limit the statistical power and generalizability of the sensory results. Although Kendall’s W (0.84, p < 0.01) indicated good inter-rater agreement, future studies should employ larger and more demographically diverse panels to confirm these findings.

**TABLE 2:** α-diversity indices of microbial communities in CTLs (mean ± SD, n = 3). Different superscript letters within the same column indicate significant differences at p < 0.05 (Kruskal–Wallis test followed by Dunn’s *post hoc* test with Benjamini–Hochberg FDR adjustment). Groups sharing the same letter are not significantly different.

Group	Shannon index	Simpson index	Chao1 richness
CK	3.45 ± 0.18c	0.89 ± 0.02c	256.4 ± 18.3c
TDH	4.38 ± 0.22b	0.94 ± 0.01b	312.7 ± 20.5b
THP	4.61 ± 0.19a	0.96 ± 0.01a	338.2 ± 22.1a
TPE	4.15 ± 0.20b	0.93 ± 0.01b	298.5 ± 19.7b
TQM	4.82 ± 0.21a	0.97 ± 0.01a	356.8 ± 24.3a

**FIGURE 4:**
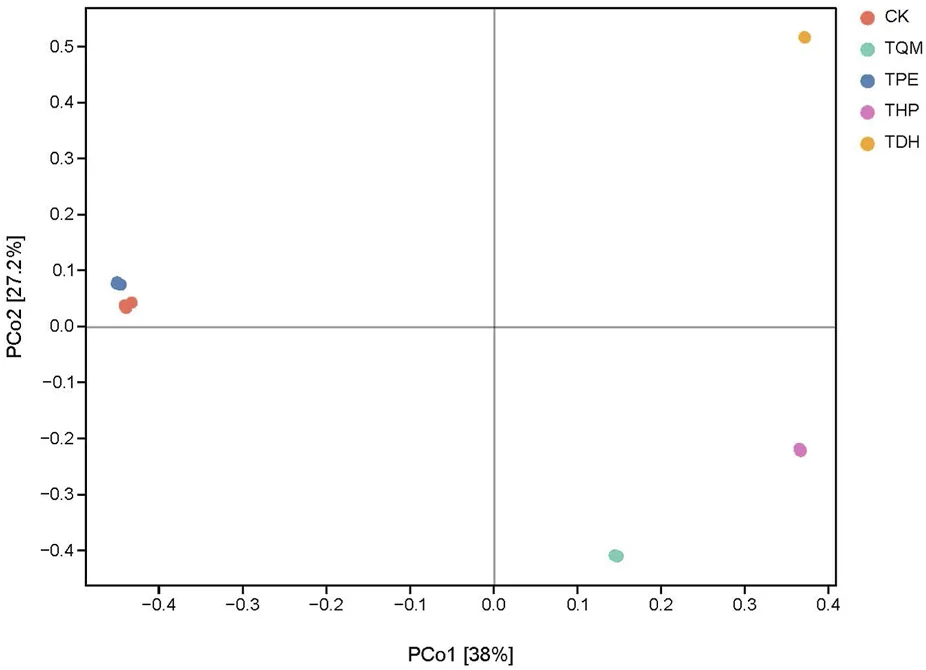
β-diversity of microbial communities in CTLs, visualized by principal coordinate analysis (PCoA) based on Bray–Curtis distances. Each point represents one independent fermentation batch (n = 3 per group). Ellipses represent 95% confidence intervals. PERMANOVA: R^2^ = 0.38, p < 0.001. Betadisper test: p = 0.42, indicating homogeneous dispersion among groups.

The microbial community composition of CTLs is shown in Figure 5. At the phylum level (Figure 5a), Proteobacteria, Firmicutes, Actinobacteriota, and Bacteroidota dominated all groups, but their relative abundances differed: BTE-treated groups had higher proportions of Proteobacteria and Actinobacteriota, whereas CK had more Firmicutes. At the genus level (Figure 5b), CK was dominated by *Pantoea*, *Staphylococcus*, *Enterobacter*, and *Sphingomonas*. BTE-treated groups showed differential enrichment. *Pantoea* was notably more abundant in TDH and TPE. *Pantoea* has been reported as a diazotrophic and plant-growth-promoting genus capable of producing indole-3-acetic acid and solubilizing phosphate (Asaf et al., 2020; Chen et al., 2017). In tobacco fermentation, *Pantoea* has been associated with the degradation of macromolecules and the production of volatile precursors (Zheng et al., 2022a). However, it should be noted that some *Pantoea* species can also be opportunistic pathogens, and their role in CTL fermentation may be strain-dependent. *Sphingomonas*, a genus with antioxidant and aroma-synthesis capabilities, was enriched in TQM (Zheng et al., 2022b; Kim et al., 2019). *Enterobacter* was significantly enriched in THP. Notably, *Staphylococcus* abundance was significantly lower in all BTE-treated groups than in CK (p < 0.05), likely due to the antibacterial activity of tea polyphenols and flavonoids (Zhang et al., 2021; Zhang et al., 2024). These findings align with previous reports that plant-derived extracts modulate microbial community structure to improve fermentation quality (Hu et al., 2025).

**FIGURE 5:**
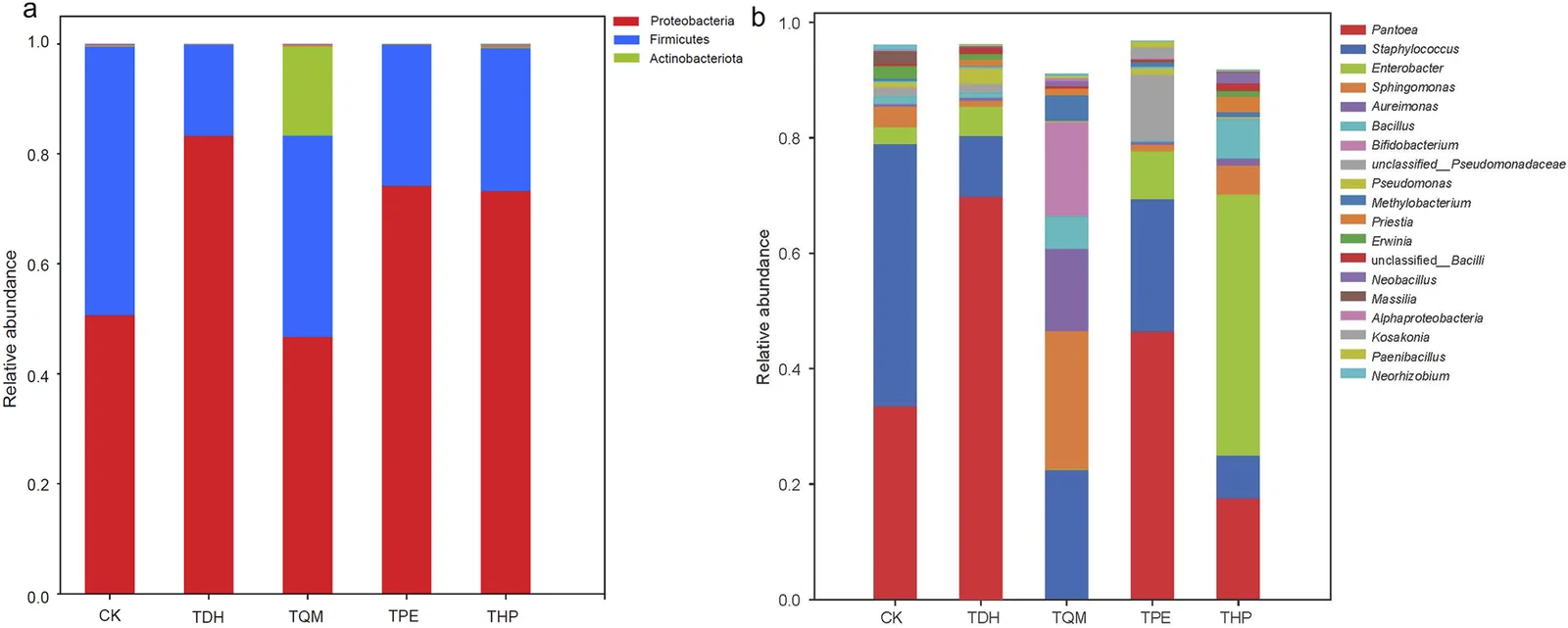
Dominant microorganisms in fermented cigar tobacco leaves. **(a)** Dominant bacterial phyla. **(b)** Dominant bacterial genera.

### Sensory evaluation

Sensory scores are summarized in Table 3. All BTE-treated groups scored significantly higher than CK in aroma richness, smoke smoothness, aftertaste, and overall quality, and significantly lower in irritancy (p < 0.05). TQM received the highest aroma richness score (8.7 ± 0.3), followed by THP (8.5 ± 0.2), consistent with their superior VAC diversity and abundance. CK had the highest irritancy (6.2 ± 0.4), while TPE had the lowest (3.1 ± 0.2), likely because tea polyphenols and amino acids bind to irritants such as aldehydes and phenols. THP scored highest in smoke smoothness (8.3 ± 0.2), followed by TQM (8.1 ± 0.3), attributable to esters and alcohols. TDH had the highest aftertaste score (7.9 ± 0.3), followed closely by THP (7.8 ± 0.3) and TQM (7.7 ± 0.3). THP achieved the highest overall quality (8.6 ± 0.2), indicating that Dahongpao extract had the most comprehensive positive effect. These results confirm that BTEs significantly enhance the sensory properties of fermented CTLs, meeting consumer preferences for low irritation, rich aroma, and smooth smoke. However, the sensory panel size (n = 5) is relatively small, which may limit the statistical power and generalizability of the sensory results. Future studies should employ larger and more demographically diverse panels to confirm these findings.

**TABLE 3:** Sensory evaluation scores of fermented CTLs (Data are presented as mean ± standard deviation (SD, n = 5 panelists × 3 replicates). Groups sharing the same letter are not significantly different (p > 0.05). Lower irritancy scores indicate less irritation.

Group	Aroma	Irritancy	Smoke smoothness	Aftertaste	Overall quality
CK	6.1 ± 0.3c	6.2 ± 0.4a	5.8 ± 0.3c	5.6 ± 0.2c	5.9 ± 0.2c
TDH	7.8 ± 0.2b	3.5 ± 0.3b	7.5 ± 0.2b	7.9 ± 0.3a	7.7 ± 0.2b
THP	8.5 ± 0.2a	3.3 ± 0.2bc	8.3 ± 0.2a	7.8 ± 0.3a	8.6 ± 0.2a
TPE	7.0 ± 0.3b	3.1 ± 0.2c	7.3 ± 0.3b	7.2 ± 0.2b	7.5 ± 0.3b
TQM	8.7 ± 0.3a	3.4 ± 0.3bc	8.1 ± 0.3a	7.7 ± 0.3a	8.4 ± 0.2a

## Discussion

### Correlations between BTE constituents and microorganisms

Since the BTEs were filtered through 0.22-μm membranes prior to application, the introduction of viable exogenous tea-associated microorganisms was excluded. Therefore, the microbial community shifts observed in BTE-treated CTLs can be primarily attributed to the chemical constituents of the extracts rather than to microbial inoculation. Tea polyphenols may act through multiple mechanisms, including disruption of bacterial membrane integrity via chelation of membrane-associated Ca^2+^ and Mg^2+^ ions, inhibition of extracellular enzyme activity, interference with quorum-sensing signaling, and generation of hydrogen peroxide under aerobic conditions (Zhang et al., 2021; Zhang et al., 2024). These mechanisms may differentially affect Gram-positive versus Gram-negative bacteria, potentially explaining the selective suppression of *Staphylococcus* (Gram-positive) while permitting the enrichment of *Sphingomonas* (Gram-negative). In parallel, tea polysaccharides and free amino acids may serve as growth substrates for specific beneficial taxa, while tea-derived organic acids may modulate the fermentation pH. The relative contributions of these chemical classes to the observed community restructuring remain to be dissected in future studies using fractionated BTE components.

To explore how BTEs influence the microbial community, we performed correlation analysis between the top 36 BTE constituents and top 30 ASVs (Figure 6). Strong associations were observed between bioactive compounds (e.g., ferulate, theanine, corilagin) and specific ASVs. Ferulate and procyanidin B2 were positively correlated with ASV4 (*Pantoea*). Gly-Val, isocitric acid, nipecotic acid, and theanine were positively correlated with ASV33 (*Bifidobacterium*). Corilagin, kaempferol-3-O-galactoside, lonicerin, methylgallate, and nicotiflorin were positively correlated with ASV20 (*Priestia*) and ASV11 (*Bacillus*), and negatively correlated with ASV10 (unclassified Pseudomonadaceae). Sultopride hydrochloride was positively correlated with *Sphingomonas* (ASV23, ASV30) and ASV24 (*Methylobacterium*), whereas ferulate and procyanidin B2 were negatively correlated with them. Theanine was positively correlated with ASV33 (*Bifidobacterium*) and ASV14 (*Bacillus*), taxa involved in amino acid metabolism and aroma formation (Lin et al., 2023). These findings suggest that BTE constituents directly modulate key microbial taxa, establishing a foundation for improved fermentation outcomes.

**FIGURE 6:**
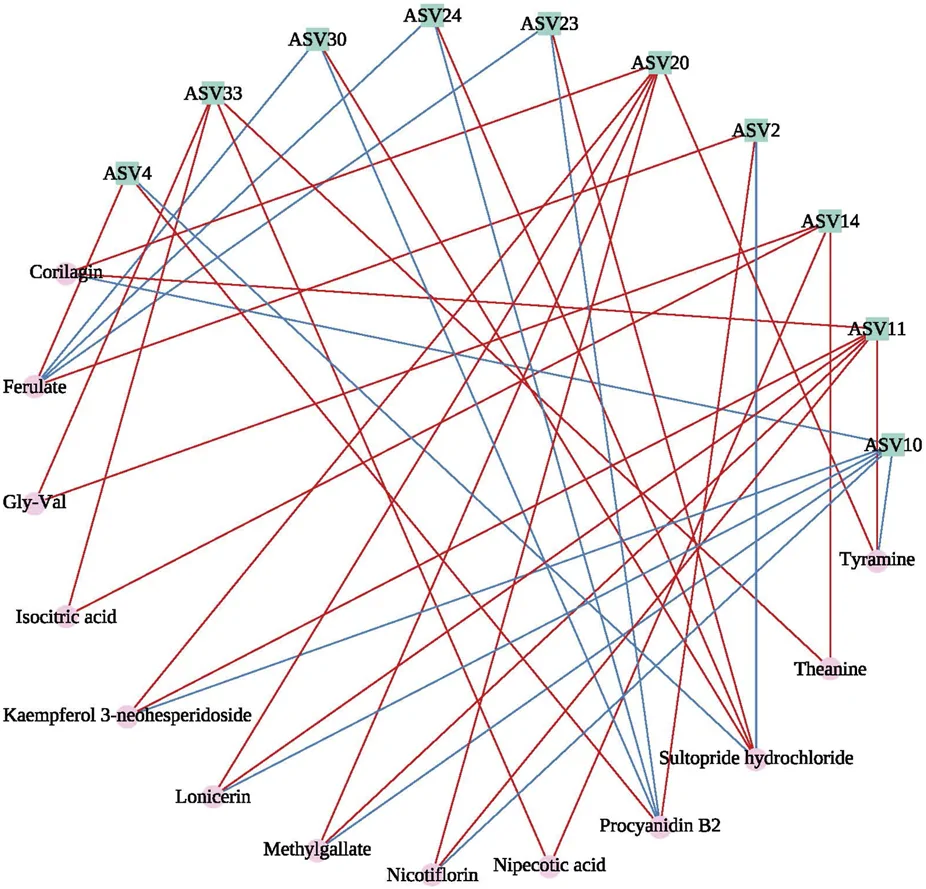
Correlation between main constituents in black tea extracts and microorganisms. Nodes represent dominant ASVs and BTE constituents; edges indicate significant correlations (|r| > 0.8, FDR-adjusted q < 0.05; red, positive; blue, negative).

### Microbial interactions

Given microbial interdependencies, even direct effects on a subset of taxa can reshape the entire community. Inter-microbial correlations are shown in Figure 7. Multiple ASVs (e.g., ASV34, ASV15, ASV26) exhibited significant mutual correlations. For instance, *Pantoea* (ASV4) was negatively correlated with *Sphingomonas* (ASV23, ASV30) and *Methylobacterium* (ASV24). Unclassified Pseudomonadaceae (ASV10) was positively correlated with *Pantoea* (ASV21) and negatively with *Priestia* (ASV20) and *Bacillus* (ASV11). *Bacillus* (ASV14) was positively correlated with *Bifidobacterium* (ASV33) and negatively with *Pantoea* (ASV21, ASV16). *Sphingomonas* (ASV23) was positively correlated with *Methylobacterium* (ASV24) and negatively with *Pantoea* (ASV31, ASV17, ASV2). *Bifidobacterium* (ASV33) was negatively correlated with *Pantoea* (ASV21, ASV16). These complex interactions indicate that BTE-induced changes propagate through the microbial network, amplifying the overall community restructuring.

**FIGURE 7:**
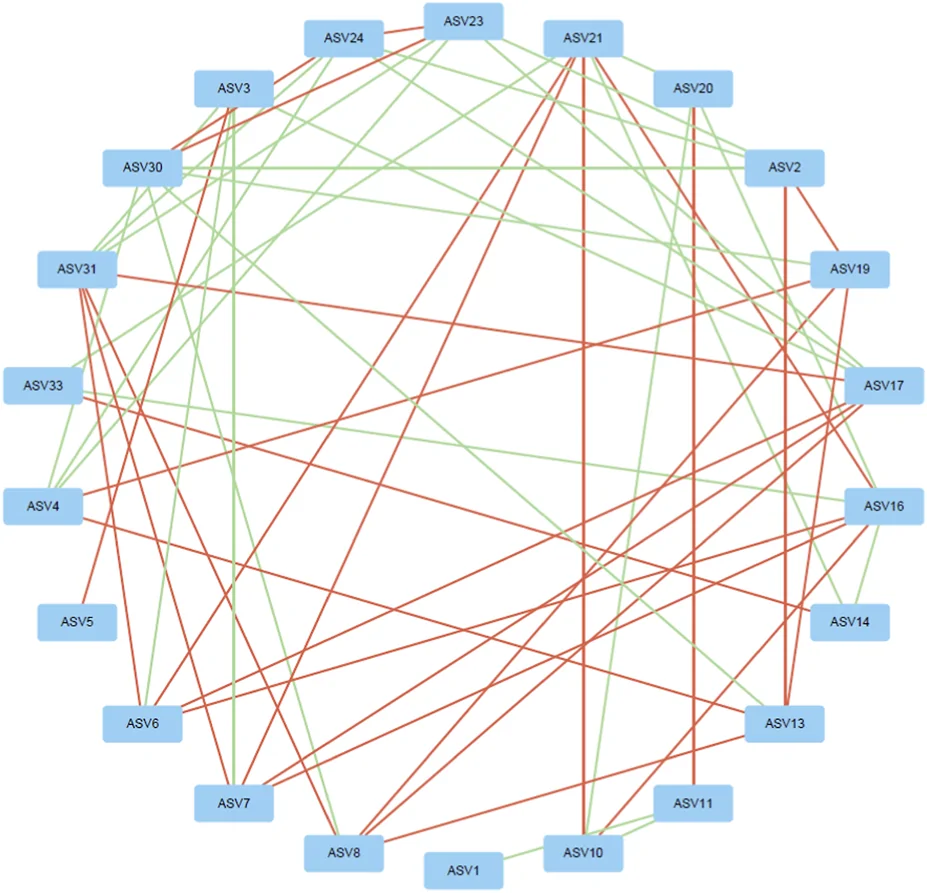
Correlation among microorganisms in cigar tobacco leaves. Nodes represent dominant ASVs; edges indicate significant correlations (|r| > 0.8, FDR-adjusted q < 0.05; red, positive; green, negative).

### Microbe–VAC relationships

The correlation between microorganisms and VACs is illustrated in Figure 8. Strong associations were found between specific ASVs and key aroma compounds. *Pantoea* (ASV4) was positively correlated with acetic acid and negatively with ethanol, hexanoic acid ethyl ester, isophorone, and 3-methylpentanoic acid ethyl ester. In contrast, *Sphingomonas* (ASV30) showed the opposite pattern. *Sphingomonas* (ASV23) and *Methylobacterium* (ASV24) were positively correlated with ethanol, hexanoic acid ethyl ester, isophorone, 3-methylpentanoic acid ethyl ester, and nonanoic acid ethyl ester, while *Pantoea* (multiple ASVs), *Bacillus* (ASV14), and *Bifidobacterium* (ASV33) were negatively correlated with these esters. *Aureimonas* (ASV12) was positively correlated with geraniol, nonanal, and o-methoxy-alpha, alpha-dimethyl-benzyl alcohol. *Enterobacter* (ASV3) was positively correlated with benzeneacetic acid ethyl ester. *Sphingomonas* (ASV18) was positively correlated with ethyl acetate. These associations indicate that specific microbial taxa drive the formation of characteristic VACs, explaining how BTEs enhance CTL aroma through microbial modulation. The sensory evaluation may have been influenced by the intrinsic aroma of the tea extracts themselves, and we cannot fully exclude the possibility that some perceived aroma enhancement originated from residual tea volatiles rather than from fermentation-derived compounds. Future studies should include a heat-treated or unfermented tea control to distinguish these contributions.

**FIGURE 8:**
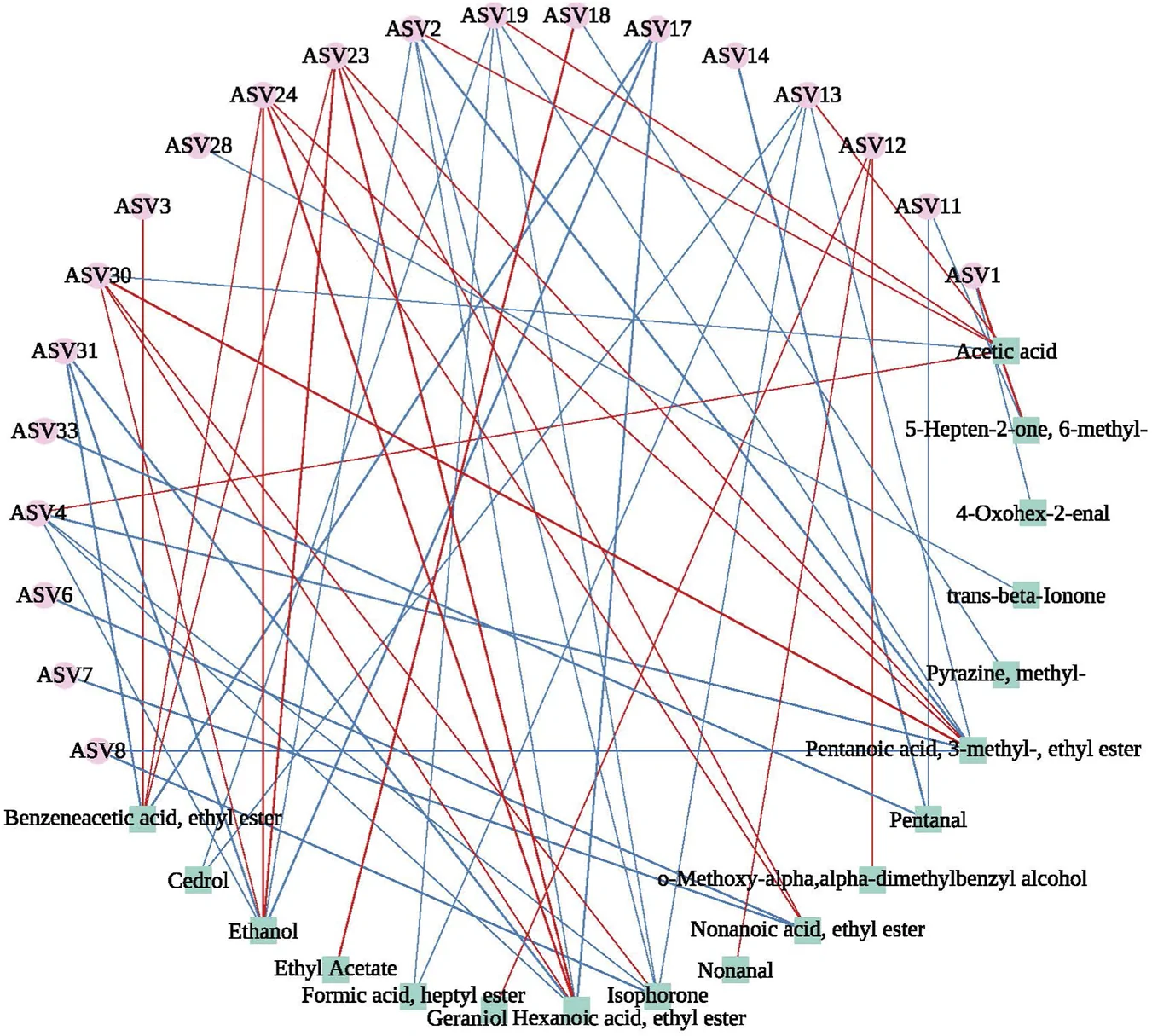
Correlation between microorganisms and volatile aroma compounds. Nodes represent dominant ASVs and VACs; edges indicate significant correlations (|r| > 0.8, FDR-adjusted q < 0.05; red, positive; blue, negative).

### Proposed regulatory mechanism

Based on these results, we propose a mechanistic model (Figure 9) for how BTEs improve CTL fermentation quality. Bioactive compounds in BTEs-particularly tea polyphenols and flavonoids-act as selective modulators, promoting potentially beneficial microbes such as *Sphingomonas* while suppressing harmful taxa like *Staphylococcus*. Other factors, including the relative abundance of other microbial taxa (e.g., *Bifidobacterium*) and the specific VAC profiles, likely contribute to the overall sensory outcome. This underscores the complexity of the fermentation ecosystem and the need for multi-factor analyses rather than single-taxon correlations. We hypothesize that acidic components in tea extracts may lower the fermentation pH, potentially favoring acid-tolerant genera such as *Bifidobacterium*. However, pH was not measured in this study, and this proposed mechanism requires direct experimental validation through pH monitoring and controlled pH-adjusted fermentation experiments. Tea polysaccharides and amino acids may serve as nutrient sources, potentially stimulating microbial metabolic activity and the production of extracellular enzymes involved in the degradation of macromolecules. However, direct measurements of enzyme activities were not performed in this study, and this proposed mechanism requires experimental validation. These collective effects reshape the microbial community into a more efficient fermentation consortium, driving the changes in volatile profiles and ultimately improving sensory quality. It should be emphasized that the proposed mechanism in Figure 9 is hypothetical and based on correlation evidence. Direct validation through pH monitoring, enzyme activity assays, and *in vitro* antibacterial tests against isolated CTL-associated strains is warranted in future studies.

**FIGURE 9:**
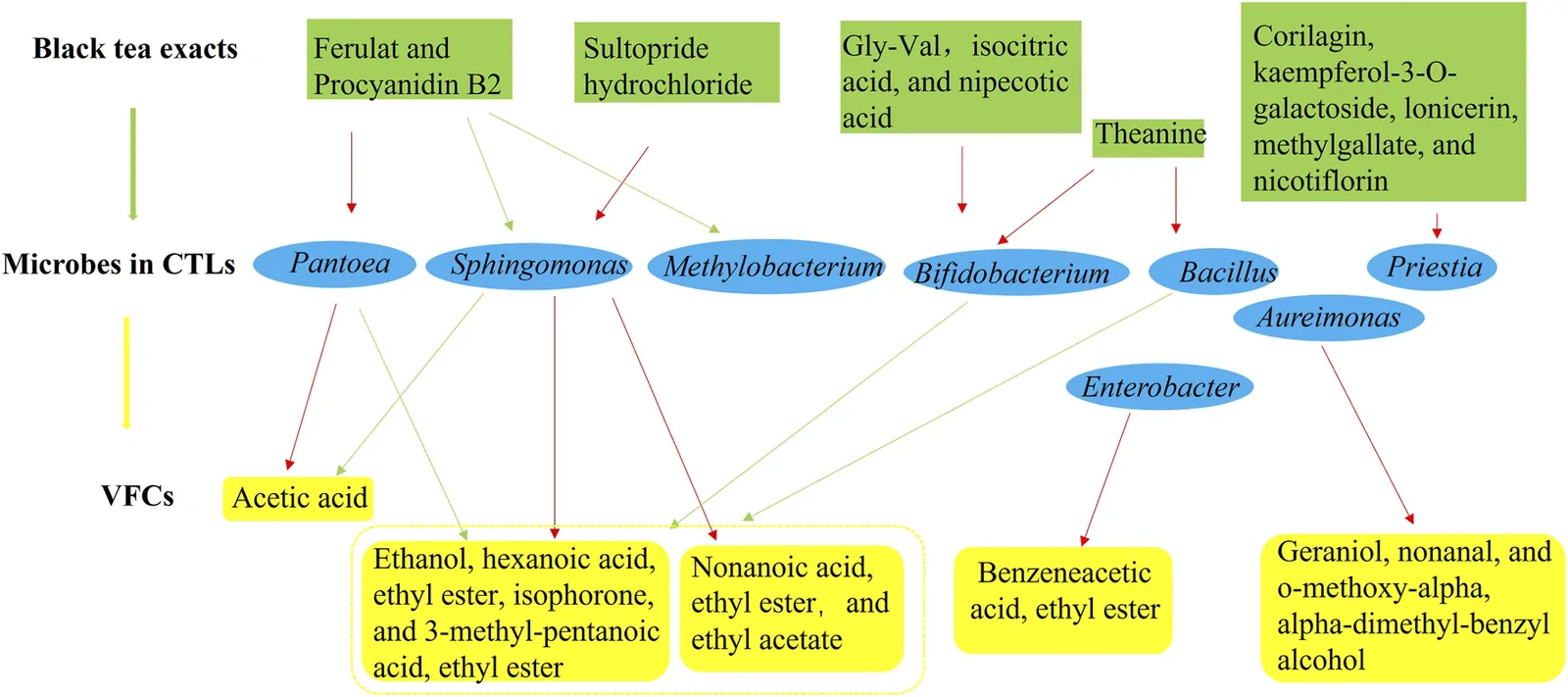
Proposed regulatory mechanism of black tea extracts on microorganisms and volatile aroma compounds in cigar tobacco leaves. Red arrows indicate positive effects; green arrows indicate negative effects. It should be noted that *Pantoea* is presented as a correlation-based candidate taxon; its functional role may be strain-dependent and requires validation through isolate-based assays.

### Comparative analysis with previous fermentation studies

The magnitude and specificity of BTE-mediated microbial modulation differ from those reported for other plant-derived additives. Compared with the citrus flavonoid treatment reported by Hu et al. (2024), which primarily enhanced *Bacillus* and reduced *Staphylococcus*, our BTE treatments induced a more pronounced enrichment of *Sphingomonas* and *Bifidobacterium*. Unlike the tobacco flower bud extract of Yao et al. (2025), which primarily suppressed *Staphylococcus* without substantially enriching specific beneficial genera, BTEs were associated with simultaneous changes in multiple taxa. However, it should be emphasized that these comparisons are based on separate studies with different experimental conditions, and direct side-by-side comparisons are needed to establish whether BTEs offer a distinct advantage. These comparisons suggest that the chemical diversity of BTEs—encompassing polyphenols, polysaccharides, alkaloids, and amino acids—confers a multifunctional modulation capacity that may be superior to single-component or less complex additives for comprehensive fermentation improvement.

It should be acknowledged that this study employed a single concentration for each BTE. While the 30% concentration was selected based on preliminary screening among four concentrations (10%–40%), this range may not fully capture the optimal dosage for each of the four tea extracts, given their distinct chemical profiles. For instance, the higher polyphenol content in Dianhong extract might require lower application rates to avoid excessive astringency, while Pu-erh extract with its relatively mild composition might benefit from higher concentrations. Future studies should conduct comprehensive dose–response optimization for each BTE individually to establish extract-specific optimal application rates.

It should also be noted that mold contamination in this study was assessed only by visual inspection. Although no visible mold growth was observed at the 30% concentration, visual inspection alone cannot exclude the possibility of mycotoxin formation by non-visible fungal colonization. Future studies should incorporate mycological enumeration (e.g., fungal plate counts) and mycotoxin analysis (e.g., aflatoxin, ochratoxin) to confirm the microbiological safety of the BTE-assisted fermentation process.

Furthermore, all analyses in this study were performed at a single time point (day 7) based on preliminary experiments that indicated stabilization of sensory attributes by this time. Nevertheless, the absence of time-series sampling limits our ability to capture the dynamic succession of microbial communities and the gradual accumulation of VACs during fermentation. The regulatory mechanisms inferred from endpoint data may not fully reflect the temporal sequence of microbial interactions and metabolite production. Future investigations should incorporate multi-timepoint sampling (e.g., days 0, 1, 3, 5, and 7) to elucidate the kinetic patterns of community assembly and aroma formation, which would strengthen causal inferences regarding microbe–metabolite relationships.

For industrial translation, several practical considerations warrant attention. First, the consistency of BTE composition across tea batches must be ensured through standardized extraction protocols and quality control measures. Second, the spray application method used in this laboratory-scale study requires adaptation to large-scale fermentation systems, potentially via automated humidification–inoculation equipment. Third, regulatory approval for tea extract application in tobacco products would be required in different jurisdictions. Despite these challenges, the natural origin and low cost of tea by-products render this strategy economically attractive compared to synthetic additive alternatives.

Comparison with conventional long-term spontaneous fermentation. The 7-day BTE-assisted fermentation employed in this study represents a substantial reduction compared to conventional CTL fermentation. However, direct comparative data between 7-day BTE-assisted and traditional long-term fermentation are not available in this study. The sensory scores achieved by BTE-treated CTLs (overall quality: 7.5–8.6) are comparable to those reported for conventionally fermented cigars in previous studies (Zheng et al., 2022a; Yang et al., 2024), suggesting that BTE supplementation may accelerate the fermentation process without compromising sensory quality. Nevertheless, prolonged fermentation may generate additional complex flavor compounds through slow Maillard reactions and microbial secondary metabolism that cannot be fully replicated in a 7-day process. Future studies should directly compare BTE-assisted short-term fermentation with traditional long-term fermentation to determine whether the accelerated process achieves equivalent quality.

## Conclusion

This study evaluated the effects of four BTEs on CTL fermentation quality under controlled laboratory conditions. BTEs—rich in tea polyphenols, theanine, and flavonoids—were associated with shifts in the microbial community, including increased Shannon diversity and altered relative abundances of specific genera. These microbial changes co-occurred with increased diversity and relative abundance of VACs and with improved sensory scores. Among the four extracts, Dahongpao and Keemun black tea exhibited the most comprehensive sensory benefits, with Keemun showing the greatest enhancement in aroma diversity, consistent with its highest VAC diversity and abundance. As natural fermentation regulators, BTEs have broad application prospects in the cigar industry. This research provides a novel technological approach for high-quality cigar production and promotes cross-industry synergy between the tea and tobacco sectors.

## Data Availability

The datasets presented in this study can be found in online repositories. The names of the repository/repositories and accession number(s) can be found in the article/Supplementary Material.
